# Multimodal quantitative magnetic resonance imaging of the thalamus in tinnitus patients with different outcomes after sound therapy

**DOI:** 10.1111/cns.14330

**Published:** 2023-06-30

**Authors:** Qian Chen, Han Lv, Zhaodi Wang, Xiaoshuai Li, Xinghao Wang, Yuyou Huang, Pengfei Zhao, Zhenghan Yang, Shusheng Gong, Zhenchang Wang

**Affiliations:** ^1^ Department of Radiology Beijing Friendship Hospital, Capital Medical University Beijing China; ^2^ Department of Otolaryngology Beijing Jingmei Group General Hospital Beijing China; ^3^ Qiyuan Lab Beijing China; ^4^ Department of Otolaryngology Head and Neck Surgery Beijing Friendship Hospital, Capital Medical University Beijing China

**Keywords:** fractional amplitude of low‐frequency fluctuations, functional connectivity, multimodal thalamic changes, sound therapy, tinnitus, voxel‐based morphometry

## Abstract

**Aims:**

This study systematically investigated structural and functional alterations in the thalamus and its subregions using multimodal magnetic resonance imaging (MRI) and examined its clinical relevance in tinnitus patients with different outcomes after sound therapy (narrowband noise).

**Methods:**

In total, 60 patients with persistent tinnitus and 57 healthy controls (HCs) were recruited. Based on treatment efficacy, 28 patients were categorized into the effective group and 32 into the ineffective group. Five MRI measurements of the thalamus and its seven subregions, including gray matter volume, fractional anisotropy, fractional amplitude of low‐frequency fluctuation, and functional connectivity (FC), were obtained for each participant and compared between the groups.

**Results:**

Patients in both the groups exhibited widespread functional and diffusion abnormalities in the whole thalamus and several subregions, with more obvious changes observed in the effective group. All tinnitus patients had abnormal FC compared with the HCs; FC differences between the two patient groups were only observed in the striatal network, auditory‐related cortex, and the core area of the limbic system. We combined the multimodal quantitative thalamic alterations and used it as an imaging indicator to evaluate prognosis before sound therapy and achieved a sensitivity of 71.9% and a specificity of 85.7%.

**Conclusion:**

Similar patterns of thalamic alterations were identified in tinnitus patients with different outcomes, with more obvious changes observed in the effective group. Our findings support the tinnitus generation hypothesis of frontostriatal gating system dysfunction. A combination of multimodal quantitative thalamic properties may be used as indicators to predict tinnitus prognosis before sound therapy.

## Graphical abstract

This study systematically investigated structural and functional alterations in the thalamus and its subregions using multimodal MRI properties, including gray matter volume, fractional anisotropy, fractional amplitude of low‐frequency fluctuation, and functional connectivity, and examined its clinical relevance in tinnitus patients with different outcomes after sound therapy. We found that similar patterns of thalamic alterations were identified in tinnitus patients with different outcomes, with more obvious changes observed in the effective group. Moreover, our findings support the tinnitus generation hypothesis of frontostriatal gating system dysfunction. A combination of multimodal quantitative thalamic properties may be used as indicators to predict tinnitus prognosis before sound therapy.

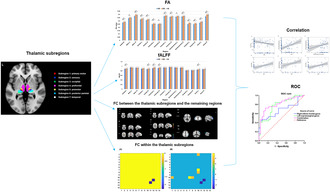

## 1 INTRODUCTION

Tinnitus, a phantom sound occurring without external sound stimulation, and its accompanying abnormalities, including distress, depression, anxiety, stress, and suicide, seriously affect quality of life.^1^, ^2^ Tinnitus, as a central nervous system abnormality, can cause significant brain reorganization closely related to patients' clinical performance and may be primarily responsible for its generation.^3^, ^4^, ^5^, ^6^ Therefore, effective and individualized therapeutic interventions are urgently needed.

Many therapeutic interventions have been used for patients with tinnitus, including counseling, pharmacological treatment, tinnitus retraining therapy, cognitive‐behavioral therapy, sound therapy, and repetitive transcranial magnetic stimulation.^7^, ^8^ However, no treatment is satisfactorily effective for all patients because the central neuropathological mechanism remains unclear.^1^, ^2^

Among these interventions, sound therapy is a cost‐effective,^9^ first‐line management for tinnitus.^10^, ^11^, ^12^ Its purpose is not to eradicate the tinnitus sound in the ears or brain but to enable adaptation to it, reduce its loudness and distress, and provide relief, ultimately improving quality of life.^13^, ^14^

Studies have focused on the therapeutic effects of sound therapy on tinnitus.^15^, ^16^, ^17^, ^18^, ^19^, ^20^, ^21^ For example, using functional and structural magnetic resonance imaging (MRI), we previously proved that sound therapy is crucial in improving the clinical symptoms of tinnitus patients by normalizing abnormal brain reorganizations; this abnormality may represent reduced involvement of the noise‐canceling system.^16^, ^17^, ^18^, ^21^ However, other studies did not observe significant improvements in symptoms after acoustic treatment,^15^, ^19^, ^20^ probably due to the low methodology quality and insufficient effect size in statistical analyses. Subsequently, we further explored central nervous system alterations in tinnitus patients with different prognoses after sound treatment.^3^, ^4^, ^22^ We found significant differences in brain structure (gray matter [GM] volume),^4^ white matter microstructure,^4^ local network connectivity,^3^ and large‐scale brain network properties^22^ at the whole brain level in patients with varying outcomes; these differences were closely related to clinical manifestations. However, these studies, from the perspective of the whole brain, only investigated possible differences in brain structure and function in patients with different curative effects.^3^, ^4^, ^22^ Few studies have systematically explored the role of core areas, including the thalamus and amygdala, in tinnitus occurrence, maintenance, and development.

As the vital station to transform and integrate sensory afferent signals,^23^ the thalamus is crucial in tinnitus generation.^6^, ^24^, ^25^ Gault et al.^24^ proposed that tinnitus occurrence and development are closely related to thalamocortical dysrhythmia (originating in the sensory thalamus), regardless of whether it is accompanied by hearing loss (HL). This abnormality exists in tinnitus, pain, and Parkinson's disease^6^ and can underpin neuropathies of these disorders.^25^ Moreover, one of our team's prior studies even found abnormal functional connectivity (FC) in tinnitus patients that can be alleviated by sound therapy.^18^ The thalamus can be divided into 14 subregions that correspond to distinct brain functions.^26^ The specific contributions of different thalamic subregions to tinnitus and its secondary dysfunction and the underlying mechanisms are undetermined, especially for tinnitus with different prognoses after sound therapy.

Here, we evaluated the prognosis of patients after 6 months of treatment and divide them into two groups: effective group (EG) and ineffective group (IG).^22^ Subsequently, we systematically investigated structural and functional alterations in the thalamus and its subregions using multimodal MRI techniques and examined their clinical relevance in tinnitus patients with different outcomes.

## 2 METHODS

### 2.1 Participants

The Ethics Committee of Beijing Friendship Hospital, Capital Medical University (No. 2017‐P2‐134‐01) approved this study. This study was conducted following the tenets of the Declaration of Helsinki. Each participant provided written informed consent before participating. The registration number on ClinicalTrials.gov is NCT03764826.

We enrolled 61 idiopathic tinnitus patients and 59 healthy controls (HCs) in this prospective observational study. Three participants (including one patient and two HCs) were excluded due to excessive head motion (>2.5 mm). Thus, the final analysis included data from 60 patients with idiopathic tinnitus and 57 HCs. Demographic data and clinical characteristics of the patients and HCs were consistent with our previous research because we used the same batch of participants in the same database.^22^ The inclusion and exclusion criteria for all participants were also consistent with our previous research.^22^

### 2.2 Sound therapy intervention and clinical evaluation

We applied a special tinnitus therapeutic instrument, eMasker® (Micro‐DSP Technology Co., Ltd.), a customized personal sound therapy device based on patient's tinnitus characteristics. The conduction process and evaluation criteria of sound therapy have been described previously.^22^ Thus, patients were divided into two groups: 28 were classified into the EG and 32 into the IG. No acoustic intervention was administered to the HCs. We calculated the Δtinnitus handicap inventory (THI) score and % improvement of THI scores of the patients, which were defined as:

$$\text{ΔTHI score}={\mathrm{THI}}_{\text{baseline}}-{\mathrm{THI}}_{\text{treated}}$$

$$\begin{array}{cc} \%\text{improvement of}\;\mathrm{THI}\;\text{scores}= & \left({\mathrm{THI}}_{\text{score}\;\mathrm{at}\;6\;\text{months follow}-\mathrm{up}}-{\mathrm{THI}}_{\text{score}\;\mathrm{on}\;\text{admission}}\right)/\\ & {\mathrm{THI}}_{\text{score}\;\mathrm{on}\;\text{admission}}\times 100\%. \end{array}$$

### 2.3 MRI protocols

All participants underwent structural and functional brain imaging using a 3.0‐T Prisma MRI scanner (Siemens); a head coil (64 channels, phased array) was used during scanning. First, axial conventional T2‐weighted imaging was performed to exclude any visible brain abnormalities. Subsequently, high‐resolution three‐dimensional (3D) T1‐weighted imaging was performed using 3D magnetization‐prepared rapid gradient‐echo sequences, diffusion tensor imaging (DTI) scanning was performed using a single‐shot gradient‐echo echo‐planar imaging (EPI) sequence, and resting‐state functional images were obtained using EPI. The scanning parameters have been described in detail previously.^4^, ^22^ The parameters for the structural scans were as follows: repetition time, 2530 ms; echo time, 2.98 ms; inversion time, 1100 ms; flip angle, 7°; total number of slices, 192; slice thickness, 1 mm; interslice gap, none; bandwidth, 240 Hz/pixel; matrix, 256 × 256; field of view, 256 × 256 mm^2^; and in‐plane resolution, 1 × 1 mm^2^. Regarding DTI, the parameters were as follows: repetition time, 8500 ms; echo time, 63 ms; matrix, 128 × 128; acquisition voxel size 2 × 2 × 2 mm^3^, field of view, 224 × 224 mm^2^, nonzero b value, 1000 s/mm^2^; gradient directions, 64; slice thickness, 2 mm; and bandwidth, 2232 Hz/Px. We obtained 74 contiguous slices. The following parameters were used for functional data: repetition time, 2000 ms; echo time, 30 ms; flip angle, 90°; total number of axial slices, 33; slice thickness, 3.5 mm; interslice gap, 1 mm; bandwidth, 2368 Hz/pixel; matrix, 64 × 64; field of view, 224 × 224 mm^2^; and in‐plane resolution, 3.5 × 3.5 mm^2^. We acquired 240 volumes.

We placed snug foam padding around the participant's head to minimize head movement during scanning. All participants were also provided with earplugs and were instructed to close their eyes but remain awake during scanning, breathe evenly, and avoid any specific thoughts.

### 2.4 Definition of the thalamic subregions

**FIGURE 1:**
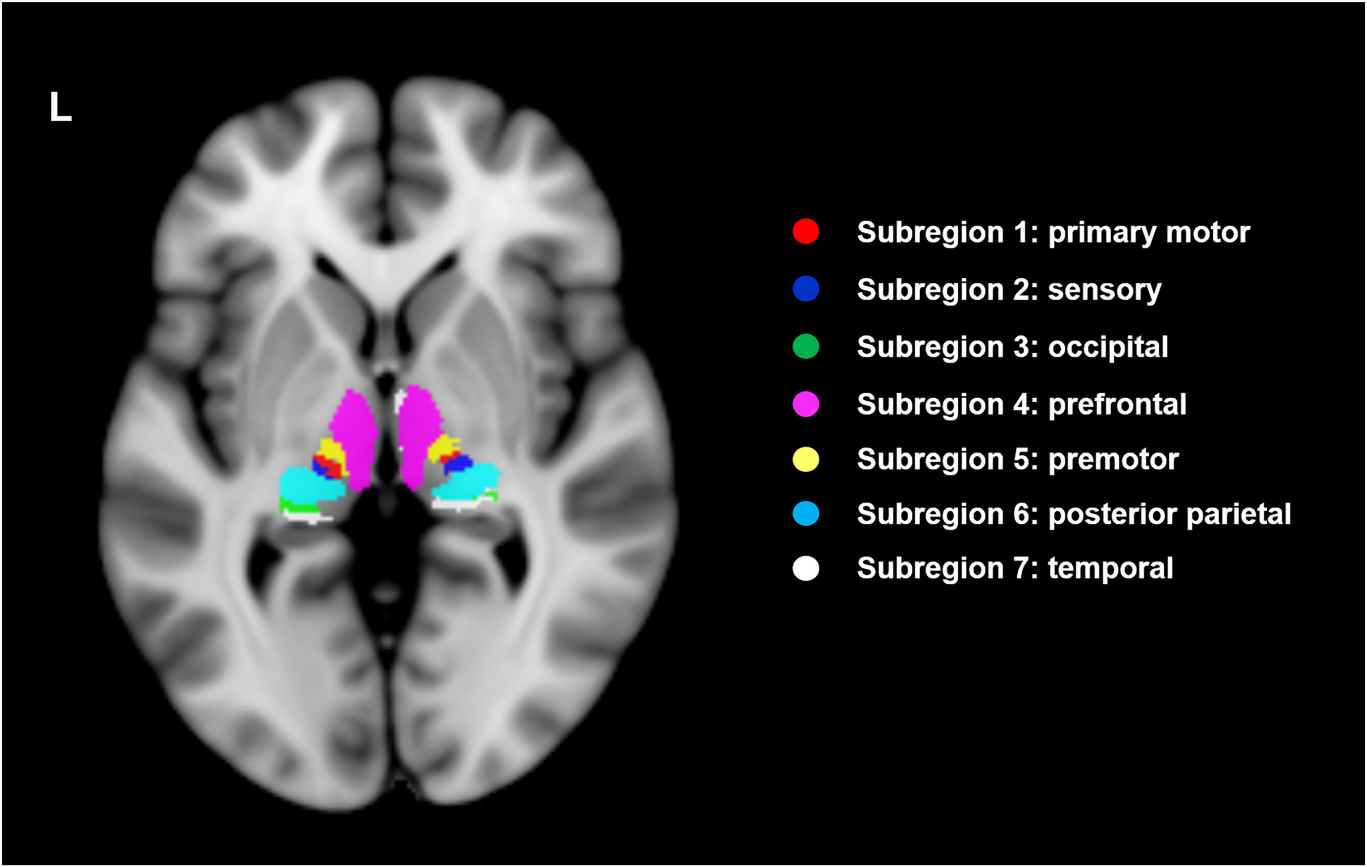
Thalamic subregions. Representative axial magnetic resonance imaging (MRI) of the thalamus subregions. A 25% threshold was used to binarize the original maximum probabilistic atlas publicly released in the FSL toolbox.

Consistent with a prior study,^26^ the thalamus was defined using a publicly available thalamic atlas in FSL 5.0.3 (Centre for FMRIB, Oxford University) (www.fmrib.ox.ac.uk/fsl). The thalamus was further divided into seven subregions (bilateral) (Figure 1). All individual multimodal maps were spatially normalized to the thalamic atlas template space.

### 2.5 Processing of structural data

All 3D high‐resolution structural data were checked to exclude scans with any artifacts, including susceptibility artifacts and artifacts due to head movement or equipment malfunction. The remaining images were preprocessed using the CAT12 package (http://www.neuro.uni‐jena.de) based on Statistical Parametric Mapping (SPM) 12: (a) segmentation, (b) normalization, and (c) smoothening. The processing steps were consistent with our previous research (see Ref. [4] for detailed information). Subsequently, the mean GM volumes of the thalamus and each thalamic subregion were extracted and compared among the three groups using analysis of covariance (ANCOVA) (SPSS version 23.0; IBM), with age and sex used as covariates. Differences between pairs of groups were evaluated using post‐hoc analysis (*p* < 0.05, uncorrected).

### 2.6 Processing of DTI data and white matter integrity property analysis

Diffusion images were processed using the PANDA toolbox (Cui et al., 2013) (http://www.nitrc.org/projects/panda/) based on FSL.^27^ The main steps were consistent with our previous research^22^: (a) converting, (b) estimating, (c) cropping, (d) co‐registration, (e) correction, (f) averaging, and (g) calculating diffusion tensor metrics. The fractional anisotropy (FA) calculation process involved preprocessing, spatial normalization of individual FA maps to Montreal Neurological Institute space, and smoothing (Gaussian kernel with a 6 mm full width at half maximum). The FA scalar values were obtained for each participant by averaging FA values across voxels within the whole thalamus and each thalamic subregion.

### 2.7 Processing of functional images and FC analysis

Resting‐state functional MRI data were processed using the Data Processing & Analysis for (Resting‐State) Brain Imaging (DPABI; http://www.rfmri.org/dpabi).^28^ The preprocessing procedure was detailed in one of the first author's previous studies.^29^ Briefly, the main steps included the following: (a) removal of the first 10 volumes, (b) slice‐timing, (c) realignment, (d) normalization, (e) smoothening, (f) regression, and (g) filtering. FC analysis was performed using the thalamus and each thalamic subregion as seeds. We performed correlation analyses within the 14 thalamic subregions and between the 15 seeds (including the whole thalamus) and the remaining voxels in the whole brain. The Fisher r‐to‐z transformation was applied to improve normality. Differences in FC among the three study groups were analyzed using the general linear model in SPM12, with total intracranial volume, age, and sex serving as covariates (voxel‐level uncorrected *p* < 0.0001; cluster‐level *p* < 0.05 with family‐wise error correction). FC differences in pairs among the groups were evaluated using post‐hoc analysis (*p* < 0.05, family‐wise correction).

### 2.8 Fractional amplitude of low‐frequency fluctuations calculation

The fractional amplitude of low‐frequency fluctuation (fALFF) calculation was consistent with that in a previous study by the first author.^30^ After preprocessing functional data (excluding filtering), fALFF was calculated using DPABI. The main steps involved transformation, calculation of the square root of the power spectrum, standardization, and calculation of the ratio of the power spectrum of the ALFF range (0.01–0.08 Hz) to the power spectrum of the entire frequency range (0–0.25 Hz) to obtain fALFF. Subsequently, the mean fALFF value of the thalamus and each thalamic subregion were extracted and compared among the three groups using ANCOVA as well.

### 2.9 Statistical analysis

We first tested the homogeneity and normality of the variance of data before analyzing the extracted values. We assessed all the data for normality using the Kolmogorov–Smirnov test. If the data were not normally distributed, nonparametric tests were used. One‐way ANCOVA and post‐hoc analysis were performed for normally distributed data and the Kruskal–Wallis H test for non‐normally distributed data. The possible relationship between the multimodal quantitative thalamic imaging properties and clinical features of tinnitus patients was explored using partial correlation analysis after adjusting for age and sex. The statistical threshold was set at *p* values <0.05.

## 3 RESULTS

### 3.1 Demographic and clinical characteristics

Table 1 summarizes the demographic characteristics of the groups. Among the three groups, there were no significant differences in age, sex, handedness, disease duration, or laterality of tinnitus. We also obtained THI scores before and after sound therapy and changes and improvements in THI scores. The EG showed significantly greater improvement in THI scores than the IG (52.30% vs. −15.79%). Moreover, we found no significant difference between the EG and IG in anxiety and depression measurements.

**TABLE 1:** Demographic and clinical characteristics.

Demographic	EG (baseline, *n* = 28)	EG (treated, *n* = 28)	IG (baseline, *n* = 32)	IG (treated, *n* = 32)	HCs (*n* = 57)	*p*‐Value
Age (years)	46.44 (±12.11)		48.54 (±12.78)		47.44 (±12.08)	0.755^a^
Sex (male/female)	12/16		22/10		31/26	0.128^b^
Handedness (right/left)	28/0		32/0		57/0	>0.99^a^
THI score	68.86 (±21.74)	33.57 (±20.16)	43.13 (±19.52)	47.88 (±20.24)	NA	<0.0001^c^
ΔTHI score	35.29 (±18.23)		−4.75 (±11.01)		NA	<0.0001^d^
% Improvement of THI score	52.30 (±19.34)		−15.79 (±42.26)		NA	<0.0001^d^
Duration (month)	≥6 & ≤48		≥6 & ≤48		NA	NA
Tinnitus pitch	250–8000 Hz		250–8000 Hz		NA	NA
Laterality (right/left/bilateral)	12/3/13		8/9/15		NA	0.158^b^
SAS	41.50 (±10.04)		42.41 (±7.47)		NA	0.691^d^
SDS	47.75 (±11.23)		47.66 (±11.50)		NA	0.975^d^

Abbreviations: EG, effective group; HCs, healthy controls; IG, ineffective group; NA, not applicable; SAS, Self‐Rating Anxiety Scale; SDS, Self‐Rating Depression Scale; THI, Tinnitus Handicap Inventory, ΔTHI score, THI_baseline_ − THI_treated_.

^a^ One‐way ANOVA.

^b^ Chi‐square test.

^c^ Two‐way ANOVA.

^d^ Two‐sample *t*‐test.

### 3.2 Morphological changes in the thalamus and thalamic subregions

**FIGURE 2:**
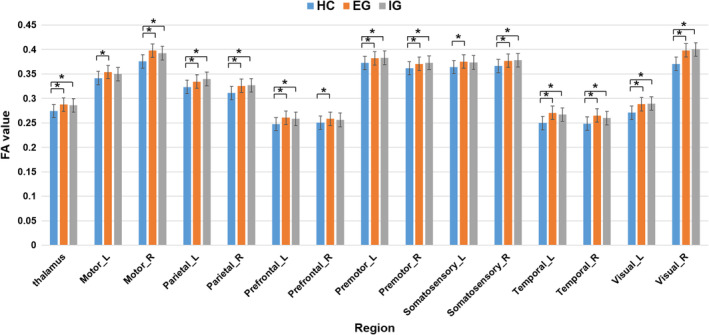
Changes in the white matter integrity properties of the thalamus and thalamic subregions. Comparisons among the three groups showed significant changes in white matter integrity properties across the entire thalamus and thalamic subregions, with only the EG showing increased FA value in the Motor_L, Prefrontal_R, and somatosensory_L compared with healthy controls (*p* < 0.05, uncorrected). EG, effective group; FA, fractional anisotropy; HC, healthy control; IG, ineffective group; L, left; R, right. *Indicates significant differences (*p* < 0.05, uncorrected).

No significant differences were found in GM volume for the whole thalamus or thalamic subregions among the three groups (Table 2; Figure S1). Post‐hoc analysis of white matter changes revealed a significant increase in FA in the whole thalamus and almost all thalamic subregions in the EG and IG compared with that in the HCs. However, only the EG showed significantly increased FA in the left motor area, left somatosensory area, and right prefrontal subregion (*p* < 0.05, uncorrected) (Table 2; Figure 2).

**TABLE 2:** Multimodal measures of the whole thalamus.

Metric	EG (*n* = 28)	IG (*n* = 32)	HCs (*n* = 57)	*p*‐Value
GM volume	1.31 ± 0.12	1.34 ± 0.12	1.29 ± 0.15	0.186
FA	0.29 ± 0.02	0.29 ± 0.02	0.27 ± 0.02	**0.005** ^a^ ^,^*
fALFF	0.90 ± 0.03	0.91 ± 0.03	0.89 ± 0.04	**0.021** ^b^ ^,^*

*Note*: Unless indicated otherwise, data are means ± standard deviations. * Bold values indicate p‐Value <0.05.

Abbreviations: EG, effective group; FA, fractional anisotropy; fALFF, fractional amplitude of low‐frequency fluctuation; GM, gray matter; HCs, healthy controls; IG, ineffective group.

^a^ Post‐hoc comparisons showed significant differences between EG and HCs.

^b^ Post‐hoc comparisons showed significant differences between IG and HCs.

### 3.3 Alterations in the regional activity of the thalamus and thalamic subregions

**FIGURE 3:**
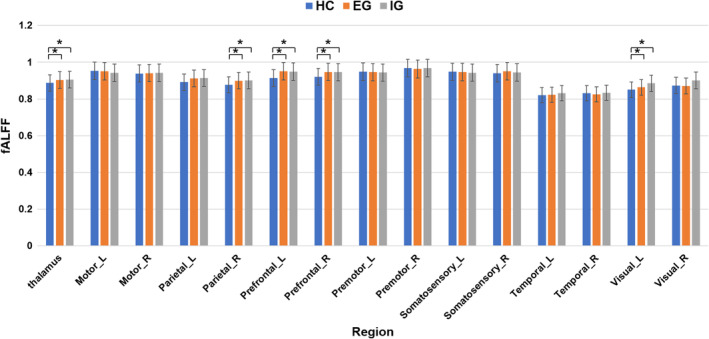
Changes in the regional activity of the thalamus and thalamic subregions. One‐way analysis of covariance showed that the fractional amplitude of low‐frequency fluctuation (fALFF) in the thalamus, Parietal_R, Prefrontal_L, Prefrontal_R, and Visual_L of the patients in the EG and IG was lower than in the HCs. No significant differences were observed in fALFF between the effective and ineffective groups. EG, effective group; HC, healthy control; IG, ineffective group; L, left; R, right. *Indicates significant differences (*p* < 0.05).

ANCOVA and post‐hoc analysis revealed that compared with the HCs, the EG and IG exhibited significantly increased fALFF in the whole thalamus, right parietal area, bilateral prefrontal region, and left visual area (*p* < 0.05, uncorrected). However, no significant differences in the mean fALFF value for other thalamic subregions were observed among the three groups (Table 2; Figure 3).

### 3.4 Alterations in FC of the thalamus and thalamic subregions

**FIGURE 4:**
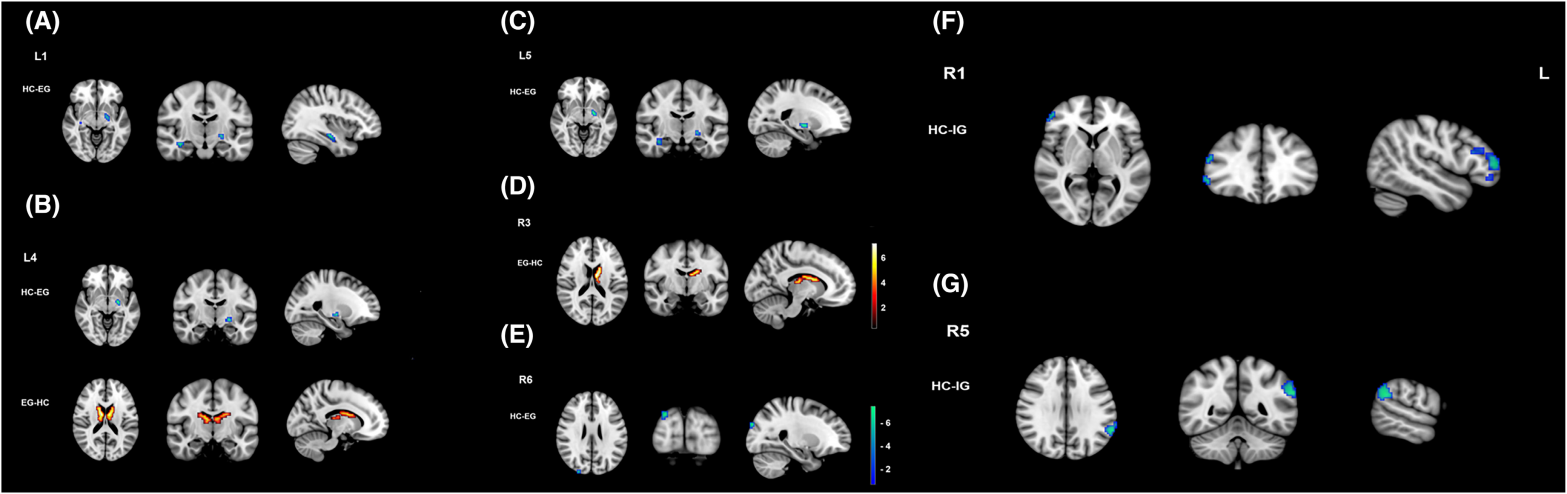
Group differences in alterations in the functional connectivity (FC) of the thalamus and thalamic subregions between the effective group (EG) and ineffective group (IG). (A) Compared with the healthy controls, the EG showed decreased FC in the left lentiform nucleus and right hippocampus when the left motor area (L1) was used as a seed. (B) The EG showed decreased FC in the left lentiform nucleus and increased FC in the bilateral thalamus when the left premotor area (L4) was used as a seed. (C) The EG demonstrated decreased FC in the left lentiform nucleus and right hippocampus when the left somatosensory area (L5) was used as a seed. (D) The EG showed increased FC in the left caudate when the right prefrontal area (R3) was used as a seed. (E) The EG demonstrated decreased FC in the right cuneus when the right temporal area (R6) was used as a seed. (F) The IG showed decreased FC in the right inferior frontal gyrus when the right motor area (R1) was used as a seed. (G) The IG demonstrated decreased FC in the left supramarginal gyrus when the right somatosensory area (R5) was used as a seed.

We found significant differences in FC among the three groups using the thalamus and thalamic subregions as seeds (detailed information see Table S1; Figure S2). Compared with the HCs, the EG showed decreased or increased FC in these brain regions: (A) left motor area (seed), left lentiform nucleus, and right hippocampus (decreased); (B) left premotor area (seed), left lentiform nucleus (decreased), and left or right thalamus (increased); (C) left somatosensory area (seed), left lentiform nucleus, and right hippocampus (decreased); (D) right prefrontal area (seed) and left caudate (increased); and (E) right temporal area (seed) and right cuneus (decreased). Conversely, the IG exhibited decreased FC in the following regions: (F) right motor area (seed) and right inferior frontal gyrus (IFG) and (G) right somatosensory area (seed) and left supramarginal gyrus (SMG) (Table 3; Figure 4). Moreover, we observed significant differences in FC within the thalamus (among the 14 thalamic subregions) among the three groups. Compared with the HCs, the EG showed increased FC between the bilateral temporal areas (Figure S3A); however, the IG showed decreased FC between the left motor and right somatosensory areas, left premotor and right somatosensory areas, and right premotor and right somatosensory areas and increased FC between the bilateral temporal regions (Figure 3B).

**TABLE 3:** Brain regions of abnormal functional connectivity with thalamic subregions between the EG and IG patients.

Brain region	Cluster size (voxels)	Peak T‐score	MNI coordinates (mm)		
			*x*	*y*	*z*
L1					
EG < HC					
Left lentiform nucleus	18	4.49	−21	−9	−6
Right hippocampus	16	4.26	36	−15	−15
L4					
EG < HC					
Left lentiform nucleus	11	4.15	−21	−9	−6
EG > HC					
Left/right thalamus	211	6.58	12	−3	18
L5					
EG < HC					
Left lentiform nucleus	15	4.41	−21	−9	−6
Right hippocampus	12	4.21	36	−15	−18
R1					
IG < HC					
Right inferior frontal gyrus	75	4.97	54	30	21
R3					
EG > HC					
Left caudate	74	5.70	−9	−3	18
R5					
IG < HC					
Left supramarginal gyrus	66	5.83	−60	−51	30
R6					
EG < HC					
Right cuneus	9	3.83	27	−93	30

*Note*: The threshold was set at a *p* < 0.05 (family‐wise error corrected).

Abbreviations: EG, effective group; HC, healthy control; IG, ineffective group.

### 3.5 Correlations between the multimodal thalamic changes and clinical features

**FIGURE 5:**
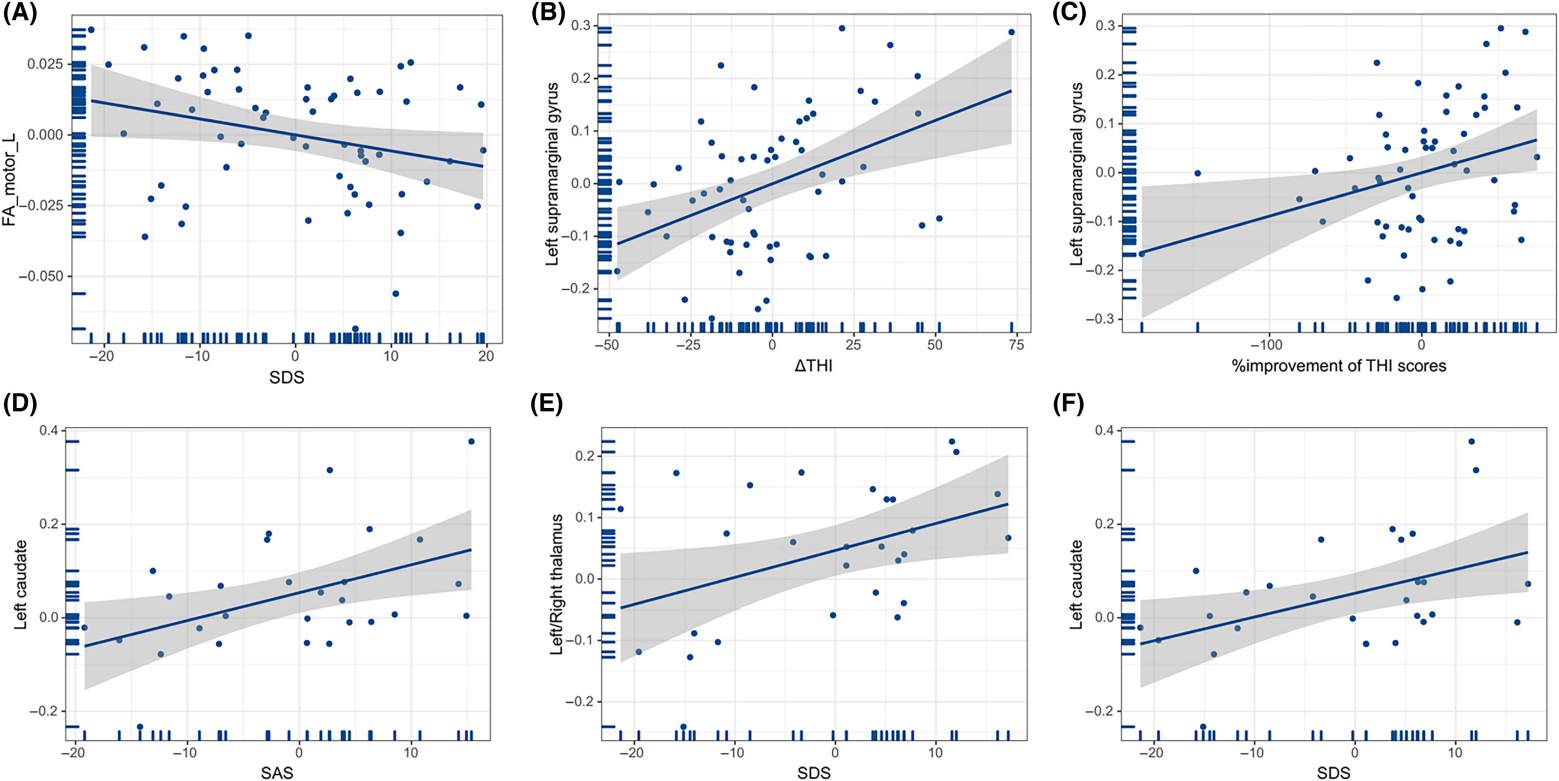
Correlations between brain imaging measurements and clinical features. In the entire tinnitus group, partial correlation analysis indicated that both Self‐Rating Depression Scale (SDS) scores were negatively correlated with the left motor area fractional anisotropy value (*r* = −0.268, *p* = 0.042; uncorrected, A) and functional connectivity (FC) strength between the right somatosensory area and the left supramarginal gyrus was positively correlated with Δtinnitus handicap inventory (ΔTHI) scores and %improvement in THI scores (*r* = 0.436, *p* = 0.001, B; *r* = 0.311, *p* = 0.017, C, separately, uncorrected). In the effective group, partial correlation analysis demonstrated that the Self‐Rating Anxiety Scale and SDS scores were positively correlated with FC strength between the right prefrontal region and left caudate (*r* = 0.481, *p* = 0.013, D; *r* = 0.454, *p* = 0.020, F; separately, uncorrected) and FC strength between the left premotor area and the left or right thalamus was positively correlated with Self‐Rating Depression Scale scores (*r* = 0.401, *p* = 0.042, uncorrected, E).

In the entire tinnitus group, partial correlation analysis indicated that Self‐Rating Depression Scale (SDS) scores correlated negatively with the left motor area FA value (*r* = −0.268, *p* = 0.042; Figure 5A) and FC strength between the right somatosensory area and the left SMG correlated positively with ΔTHI scores and % improvement in THI scores (*r* = 0.436, *p* = 0.001; *r* = 0.311, *p* = 0.017, separately, uncorrected; Figure 5B,C). In the EG, partial correlation analysis demonstrated that Self‐Rating Anxiety Scale (SAS) and SDS scores correlated positively with FC strength between the right prefrontal region and left caudate (*r* = 0.481, *p* = 0.013; *r* = 0.454, *p* = 0.020; separately, uncorrected; Figure 5D,F) and FC strength between the left premotor area and the left or right thalamus correlated positively with SDS scores (*r* = 0.401, *p* = 0.042, uncorrected; Figure 5E). No other significant correlations were discovered between clinical factors and brain imaging properties in patients in the IG (*p* > 0.05, uncorrected).

### 3.6 Combination of the multimodal thalamic alterations as imaging biomarkers

**FIGURE 6:**
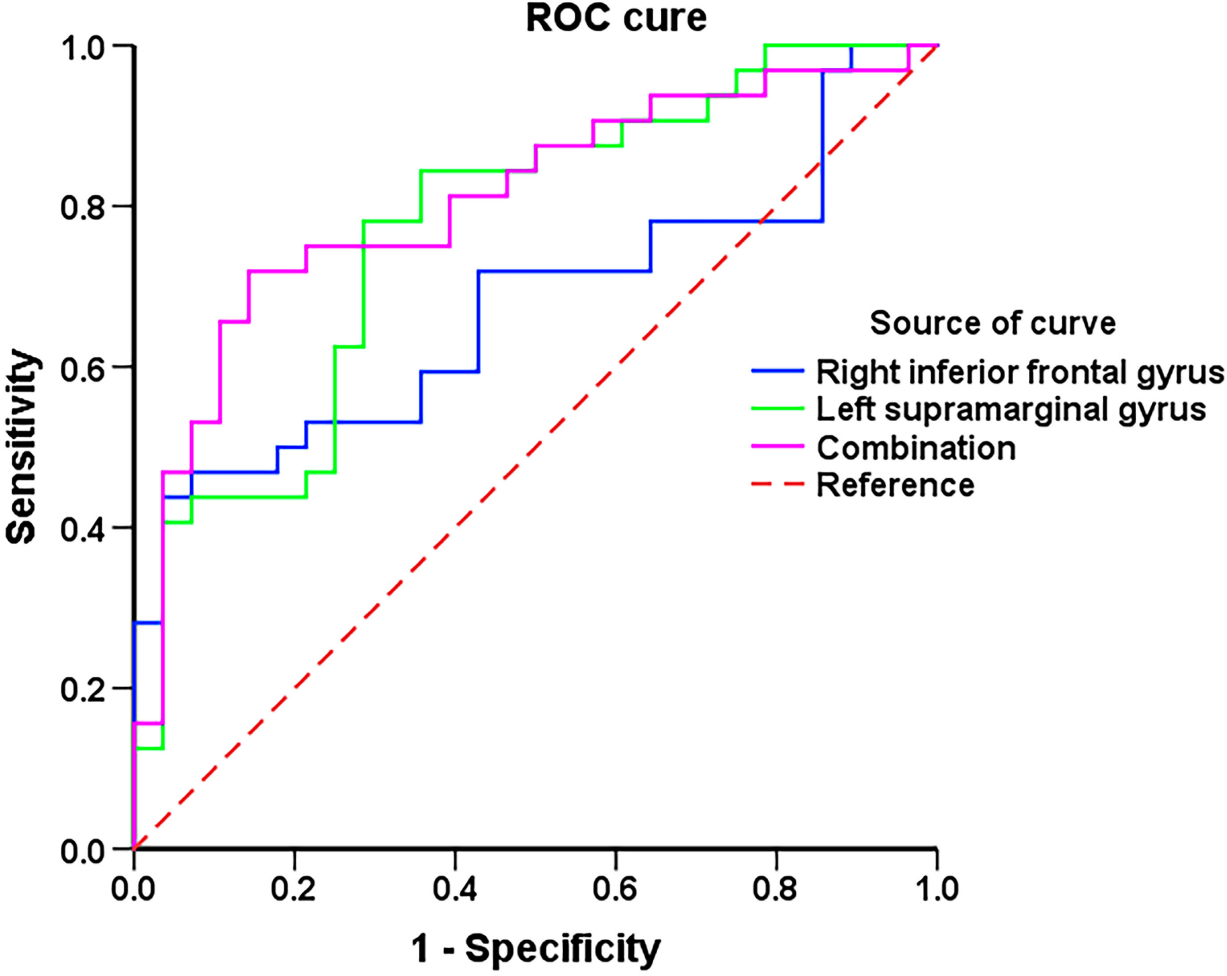
Receiver operating characteristic (ROC) curve for multimodal quantitative thalamic properties alterations as indicators. An optimal cutoff value (0.402, 0.495, and 0.576) of the exact thalamic functional imaging indicator and their combination was determined at a sensitivity of 43.8%, 78.1%, and 71.9%, and a specificity of 96.4%, 71.4%, and 85.7%, respectively. The areas under the curve for the ROC curve were 0.675, 0.770, and 0.808 (95% confidence intervals: 0.538–0.813, 0.650–0.890, and 0.696–0.920, respectively).

Figure 6 and Table 4 show the sensitivity and specificity of thalamic functional changes in tinnitus patients for prognosis evaluation and screening before acoustic treatment. Applying cutoff values of 0.402, 0.495, and 0.576, we evaluated the prognoses and screened patients with a sensitivity of 43.8%, 78.1%, and 71.9%, respectively, and a specificity of 96.4%, 71.4%, and 85.7%, respectively, using each exact thalamic functional imaging indicator or their combination, respectively. The corresponding areas under the curve (AUCs) for the receiver operating characteristic (ROC) curve values were 0.675, 0.770, and 0.808, respectively. Moreover, the positive predictive value (PPV) and negative predictive value (NPV) for each exact thalamic functional imaging indicator or their combination were 40.6% and 47.1%, 70.4% and 78.9%, and 81.5% and 77.7%, respectively. Figure S4 and Table S2 show the ROC analysis information of all thalamic structural and functional imaging alterations.

**TABLE 4:** Results of ROC curve analysis of the thalamic subregions' functional properties as prognostic indicators.

Thalamic subregions' properties	AUC	Sensitivity	Specificity	*p*‐Value	Cutoff	PPV (%)	NPV (%)
R inferior frontal gyrus	0.675	0.438	0.964	**0.02** *	0.402	40.6	47.1
L supramarginal gyrus	0.770	0.781	0.714	**0.000** *	0.495	70.4	78.9
Combination	0.808	0.719	0.857	**0.000** *	0.576	81.5	77.7

Abbreviations: AUC, area under curve; L, left; NPV, negative predictive value; PPV, positive predictive value; R, right; ROC, receiver operator characteristic.

* Bold values indicate p‐Value <0.05.

## 4 DISCUSSION

In this study, we presented structural and functional alterations of the whole thalamus and thalamic subregions in idiopathic tinnitus with different outcomes using multimodal quantitative MRI techniques. Structurally, the EG and IG exhibited white matter microstructural abnormalities (increased FA values) across the entire thalamus; however, these changes were more obvious in the EG. Functionally, decreased integrations within the thalamus and between the thalamus and other regions were detected in the EG and IG; the former is mainly found in the IG and the latter in the EG. Moreover, we discovered that improving clinical symptoms in tinnitus patients (mainly in the EG) was closely associated with alterations in FC between the thalamus and other brain areas (left SMG, thalamus, and left caudate). These findings supported the idea that the thalamus is crucial in tinnitus and provided new insight into the possible mechanisms underlying the different effects of sound therapy.

### 4.1 Morphological and regional activity changes in the thalamus and thalamic subregions

In our study, we discovered increased FA in almost the entire thalamus in the EG and IG. Benson et al.^31^ considered FA as a potential local marker of aberrant or increased neural connectivity. Thus, increased FA in thalamic radiation in tinnitus may reflect a new steady state of functional and anatomical connectivity induced by peripheral end organ‐induced damage/deafferentation. Moreover, Jaroszynski et al.^32^ reported that thalamo‐parietal bundles displayed tinnitus‐related increased apparent fiber density and FA. They believed that these tinnitus changes possibly reflect increased connectivity with auditory areas, directly affecting tinnitus perception. Consistent with these studies, the increased FA observed in our study could be a secondary, compensatory alteration in neural connectivity between the thalamus and the auditory and other limbic areas, resulting in a new stable state. Moreover, comparing the increased FA in the left motor and right prefrontal subregions in the EG with that in the HCs indicated more active functional integration, including auditory‐somatosensory‐emotional plasticity in tinnitus.^32^, ^33^ In addition, the negative correlation between the left motor subregion and SDS scores indicated that the more apparent white matter microstructure reorganization in this area, the less severe the clinical depression symptoms of tinnitus patients, and the exact underlying mechanism require investigations. Unlike the HCs, no thalamic GM volume changes were observed in tinnitus patients. This indicates that although the thalamus is crucial in functional integration after tinnitus, thalamic remodeling is manifested in changes in white matter microstructure and brain function.

The EG and IG exhibited abnormal fALFF in the thalamus and several subregions, including the right parietal, bilateral prefrontal, and left visual subregions. This may indicate compensatory or secondary functional remodeling of the brain after tinnitus, characterized by increased integration between the thalamus and its corresponding brain regions; the thalamus combines inputs from several brain areas, including somatosensory, auditory, visual, motor, cingulate, and prefrontal cortices, and integrates proprioceptive and vestibular signals from subcortical areas, resulting in a new stable state.^31^, ^32^ This functional reorganization may be independent of the effectiveness of treatment intervention.

### 4.2 Functional changes in the thalamus and thalamic subregions

Abnormal connectivity between the thalamic subregions and auditory and non‐auditory‐related brain areas was observed in the EG and IG. Nonetheless, significant differences were observed between these groups compared with in the HCs, mainly in the striatal network, auditory‐related cortex, and the core area of the limbic system. As core areas of the striatum or striatal network, the lentiform nucleus and caudate (especially the caudate) are crucial in tinnitus perception.^34^ When the caudate nucleus is targeted using deep brain stimulation, significant reduction in tinnitus loudness^35^ and increased connectivity with the tinnitus network were reportedly observed.^36^ Data from an animal model also proved that the caudate‐putamen nucleus plays a sensory gating role in tinnitus.^37^

The thalamus and hippocampus, as important parts of the limbic system, are closely related to tinnitus generation and are indispensable to the tinnitus frontostriatal gating system.^34^ Besides the theory that tinnitus results from thalamocortical dysrhythmia triggered by peripheral damage,^24^ scholars have discovered that decreased FC between the thalamus and auditory cortical areas in tinnitus may reflect the disrupted thalamic gating mechanism.^38^, ^39^ The auditory association cortex receives indirect input from the hippocampus via the parahippocampal or perirhinal cortex.^40^ The hippocampal‐auditory network is critical for forming long‐term auditory memories.^41^ For the SMG and cuneus, anatomically, the former mainly processes auditory information, whereas the latter is closely associated with visual function. Leaver et al.^42^ suggested that patients with larger SMG are better at incorporating tinnitus into unattended background noise, that is, they may better perceive tinnitus. Moreover, tinnitus might decrease spontaneous activity in the visual regions because of the salience of the tinnitus perception.^43^ Thus, the functional changes between the thalamus and cuneus may result from multisensory interactions between the auditory and visual regions in tinnitus.^44^ The IFG, a frontal region involved in tinnitus, serves as the core region of response inhibition; its activity might mirror the attempt to control the bottom‐up attention allocated to the tinnitus percept in a top‐down way.^45^ In addition, we discovered that the FC between the thalamic subregions and SMG, thalamus, and caudate nucleus correlated positively with tinnitus patients' negative emotional scores (SDS and SAS scores). The SMG is strongly related to tinnitus perception, whereas the latter two are vital nodes of the frontostriatal gating system. Therefore, these relationships suggest that the more abnormal the patients' tinnitus perception or the gating system, the worse the negative emotions.

The areas with abnormal FC within the thalamic subregions described above are critical brain areas involved in tinnitus generation. All these brain areas are part of the frontostriatal gating system except the SMG. In addition, although FC changes were observed in the EG and IG, the EG showed more alterations, mainly in the connectivity between the thalamus and other brain areas. Furthermore, the IG mainly involves the connectivity abnormalities within the thalamus. Thus, we speculated that this might be responsible for the different efficacies after sound therapy, and its exact mechanism requires further study.

### 4.3 Combination of the multimodal thalamic alterations as imaging biomarkers

Using the combination of the altered FC value in the right IFG and left SMG as a possible indicator, at the cutoff value of 0.576, we obtained sensitivity, specificity, AUC value, PPV, and NPV of 71.9%, 85.7%, 0.808, 81.5%, and 77.7%, respectively. The results revealed that these factors could be used as imaging indicators to screen tinnitus patients and predict prognosis before acoustic therapy.

Few studies have applied brain alterations to predict therapeutic outcomes or screen patients before sound therapy.^3^, ^4^, ^46^ In our team's prior studies, we detected that structural and functional alterations could be optimal imaging biomarkers for predicting the efficacy of sound therapy in tinnitus patients.^3^, ^4^ Moreover, Han et al.^46^ applied baseline FC features to predict clinical symptom improvements after sound therapy and found that the best indicators were properties of the bilateral thalami, with adjusted AUC values of approximately 0.8. Here, we used the combination of FC changes between the thalamic subregions, right IFG, and left SMG as an indicator to screen patients and evaluate their prognosis before sound therapy and obtained relatively optimal specificity, sensitivity, PPV, and NPV.

This study had several limitations. First, this was a cross‐sectional study with a relatively small sample size; hence, future follow‐up studies with a larger sample size must confirm the repeatability and track the distinct longitudinal changes in the thalamus as the disease progresses. Second, as a treatment‐related study, applying sham treatment to patients and HCs is necessary to eliminate the placebo effect of sound therapy. Third, a proportion of patients had different degrees of HL, and tinnitus lateralization was inconsistent. In future studies, we should eliminate the possible impact of these factors on results, including recruiting tinnitus patients without HL and those with the same sidedness. Fourth, a relatively low sensitivity (<75%) was obtained when predicting the outcome of patients with tinnitus after acoustic treatment using the ROC curve. In the future, the discriminative power could be improved using more advanced approaches (machine learning and deep learning). Finally, the brain structural and regional activity analyses were not statistically corrected, but the results demonstrated at least a trend of thalamic reorganization in tinnitus; conclusions should be verified in a larger population.

## 5 CONCLUSION

In conclusion, similar patterns of thalamic alterations were identified in tinnitus patients with different outcomes after acoustic treatment, with the EG showing more obvious changes, especially in thalamic functional alterations. Our findings support and further confirm the tinnitus generation hypothesis of frontostriatal gating system dysfunction. A combination of multimodal quantitative thalamic properties, mainly the abnormal FC alterations, may be used as potential indicators to predict tinnitus prognoses and screen patients before sound therapy.

## AUTHOR CONTRIBUTIONS

Qian Chen contributed to the study concept, design, and image data processing. Qian Chen performed the statistical analysis, interpretation, and drafting of the article. Han Lv, Zhaodi Wang, Xiaoshuai Li, Xinghao Wang, Yuyou Huang, Pengfei Zhao, Zhenghan Yang, and Shusheng Gong provided technical and clinical support. Qian Chen, Han Lv, and Zhenchang Wang contributed to the study concept, and revising of the article for intellectual content and agree to be accountable for all aspects of the work.

## FUNDING INFORMATION

This work was supported by Grant No. 61931013, 62171297, and 82171886 from the National Natural Science Foundation of China, No. [2015] 160 from Beijing Scholars Program.

## CONFLICT OF INTEREST STATEMENT

The authors declare no financial or other conflicts of interest.

## Supporting information


Figures S1–S4.

Table S1.

Table S2.

## Data Availability

The datasets generated for this study are available on request to the corresponding authors.
