# Supplementary material for: Multimodal quantitative magnetic resonance imaging of the thalamus in tinnitus patients with different outcomes after sound therapy
Source: CNS Neurosci Ther. 2023 Jun 30;29(12):4070–81. doi: 10.1111/cns.14330 (PMC10651975; doi:10.1111/cns.14330)
Supplement: Supplementary file 1 — Figures S1–S4. [file CNS-29-4070-s003.docx]

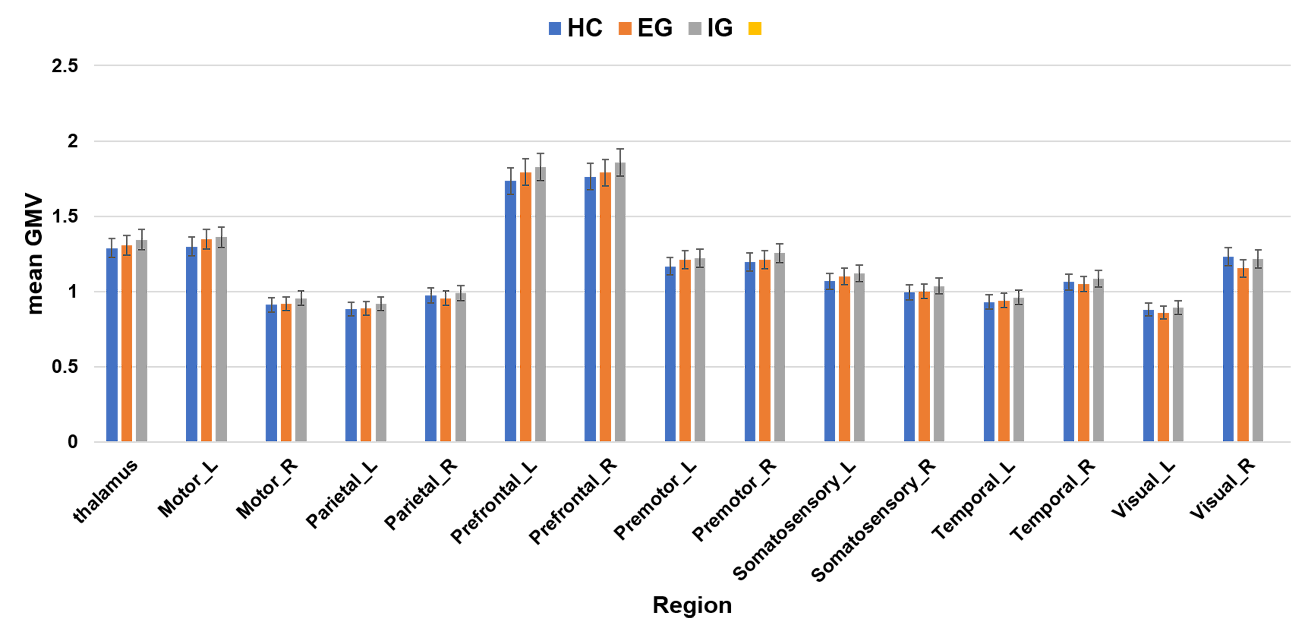


**Supplementary Figure1**


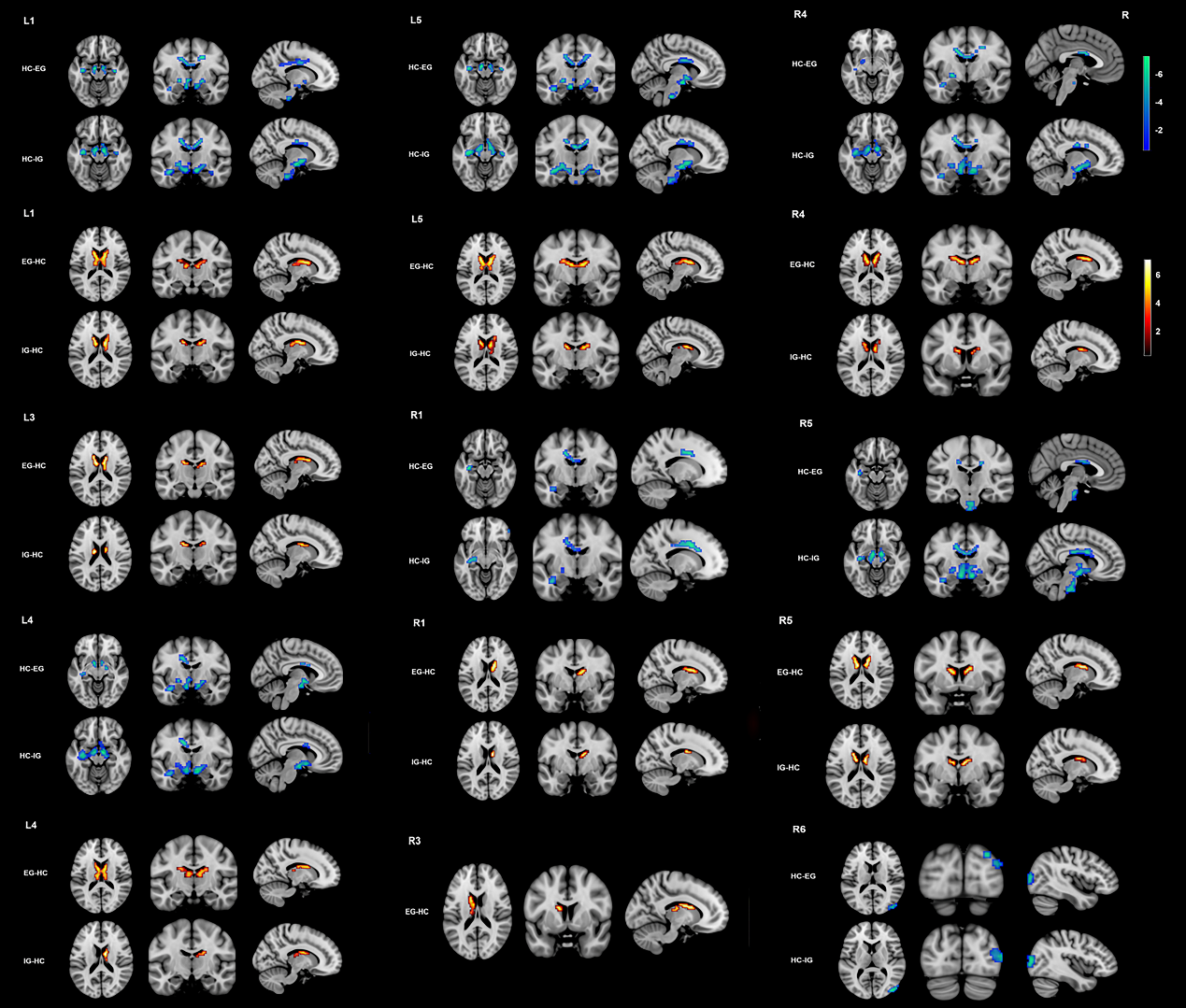


**Supplementary Figure2**


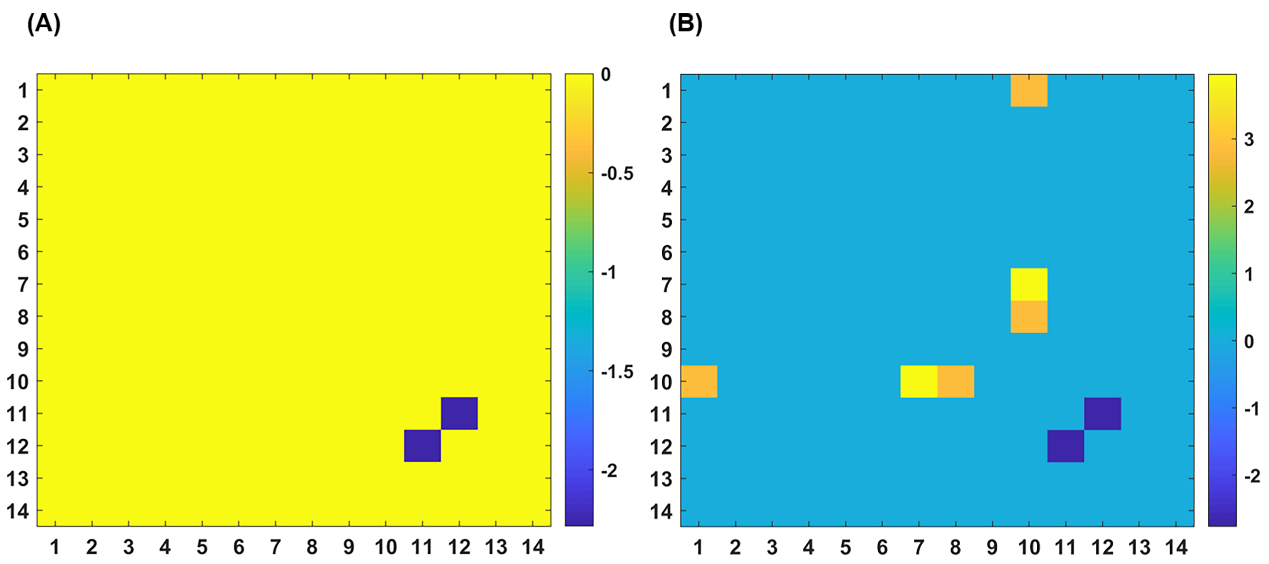


**Supplementary Figure3**


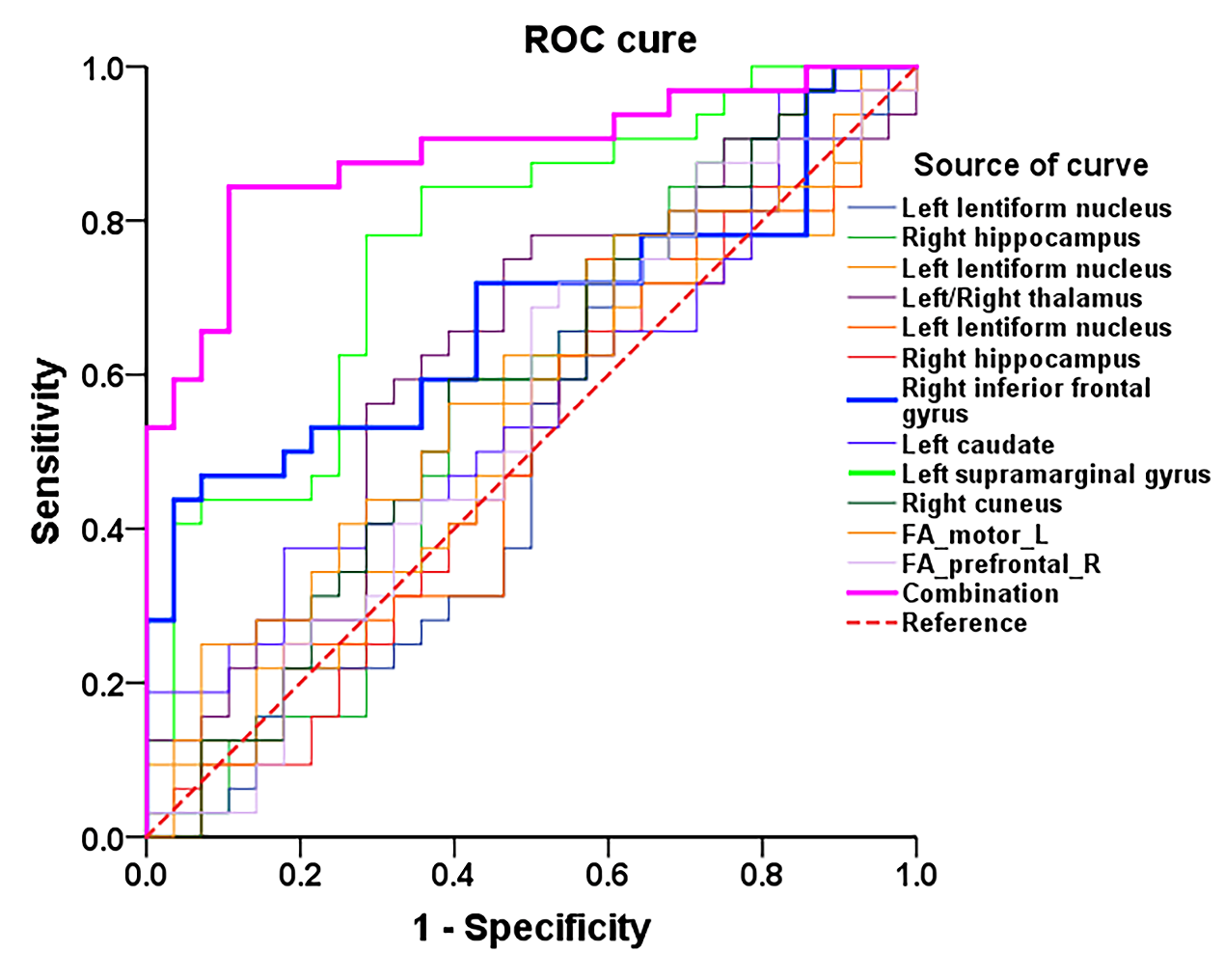


**Supplementary Figure4**

## Supplementary Figure Legends

**Supplementary figure 1.** **Changes in the gray matter volume of the thalamus and thalamic subregions**

Comparisons among the three groups showed no significant differences in gray matter volume across the entire thalamus and thalamic subregions (p> 0.05, uncorrected). L, left; R, right; EG, effective group; IG, ineffective group; HC, healthy control; GMV, gray matter volume.

*Indicates significant differences (p < 0.05, uncorrected)

**Supplementary figure 2.** **Group differences in alterations in the functional connectivity (FC) of the thalamus and thalamic subregions among the effective group (EG), ineffective group (IG), and healthy controls (HCs).**

Compared with the HCs, both the EG and IG showed decreased FC in the left hippocampus, cingulate gyrus, midbrain, and pons, and increased FC in the bilateral caudate when the L1 was used as a seed; both the EG and IG showed increased FC in the bilateral caudate when the L3 was used as a seed; both the EG and IG showed deceased FC in the left hippocampus, midbrain, cingulate gyrus when the L4 was used as a seed; both the EG and IG showed decreased FC in the cingulate gyrus, left hippocampus, midbrain, and pons and increased FC in the bilateral caudate when the L5 was used as a seed; both the EG and IG showed deceased FC in the left hippocampus and cingulate gyrus and increased FC in the right caudate when the R1 was used as a seed; both the EG and IG showed decreased FC in the left hippocampus, cingulate gyrus, left lentiform nucleus, and pons and increased FC in the bilateral caudate when the R4 was used as a seed; both the EG and IG showed decreased FC in the cingulate gyrus, pons, and left hippocampus and increased FC in the bilateral caudate when the R5 was used as a seed; both the EG and IG showed decreased FC in the right middle occipital gyrus when the R6 was used as a seed.

Compared with the HCs, the EG showed decreased FC in the left lentiform nucleus and right hippocampus when the L1 was used as a seed. The EG showed decreased FC in the left lentiform nucleus and increased FC in the bilateral thalamus when the L4 was used as a seed. The EG demonstrated decreased FC in the left lentiform nucleus and right hippocampus when the L5 was used as a seed. The EG showed increased FC in the left caudate when the R3 was used as a seed. The EG demonstrated decreased FC in the right cuneus when the R6 was used as a seed. The IG showed decreased FC in the right inferior frontal gyrus when the R1 was used as a seed. The IG demonstrated decreased FC in the left supramarginal gyrus when the R5 was used as a seed. L1, left motor area; L3 left prefrontal area, L4, premotor area; L5, somatosensory area; R1, right motor area; R3, prefrontal area; R4, right premotor area; R5, right somatosensory area; R6, right temporal area.

**Supplementary figure 3. Group differences in alterations in the functional connectivity (FC) within the thalamus between the effective group (EG) and ineffective group (IG)**

(**A**) Compared with the healthy controls, the EG showed increased FC between the bilateral temporal area; (**B**) the IG showed decreased FC between the left motor and right somatosensory areas, the left premotor and right somatosensory areas, the right premotor and right somatosensory areas, and increased FC between the bilateral temporal regions.

**Supplementary figure 4. Receiver operating characteristic (ROC) curve for multimodal quantitative thalamic properties alterations as indicators.**

An optimal cutoff value (0.138, 0.201, 0.161, 0.286, 0.179, 0.143, 0.402, 0.196, 0.495, 0.201, 0.179, 0.188, and 0.737) of the exact thalamic functional imaging indicator and their combination was determined at a sensitivity of 78.1%, 59.4%, 62.5%, 75%, 75%, 100%, 43.8%, 37.5%, 78.1%, 59.4%, 25%, 68.8%, and 84.4%, and specificity of 35.7%, 60.7%, 53.6%, 53.6%, 42.9%, 14.3%, 96.4%, 82.1%, 71.4%, 60.7%, 92.9%, 50%, and 89.3%, respectively. The areas under the curve for the ROC curve were 0.504, 0.565, 0.522, 0.625, 0.504, 0.511, 0.675, 0.566, 0.770, 0.574, 0.577, 0.544, and 0.887 (95% confidence intervals: 0.353-0.656, 0.414-0.715, 0.374-671, 0.481-0.769, 0.355-0.654, 0.361-0.661, 0.538-0.813, 0.420-0.712, 0.650-0.890, 0.426-0.721, 0.431-0.723, 0.394-0.693, 0.394-0.693, and 0.0.802–0.0.973, respectively).
