# Supplementary material for: Multimodal quantitative magnetic resonance imaging of the thalamus in tinnitus patients with different outcomes after sound therapy
Source: CNS Neurosci Ther. 2023 Jun 30;29(12):4070–81. doi: 10.1111/cns.14330 (PMC10651975; doi:10.1111/cns.14330)
Supplement: Supplementary file 2 — Table S1. [file CNS-29-4070-s002.docx]

**Supplementary table1.** **Brain regions of abnormal functional connectivity with thalamic subregions among the EG, IG patients and HCs.**

| **Brain region** | **Cluster size**  **(voxels)** | **Peak T-score** | **MNI Coordinates (mm)** | | |
| --- | --- | --- | --- | --- | --- |
|  |  |  | **x** | **y** | **z** |
| **L1** |  |  |  |  |  |
| **EG** **< HC** |  |  |  |  |  |
| Left hippocampus | 45 | 5.93 | -36 | -12 | -18 |
| Cingulate gyrus | 141 | 5.45 | -18 | -30 | 33 |
| Midbrain | 50 | 5.16 | 21 | -9 | -6 |
| Midbrain | 52 | 4.93 | -6 | -9 | -15 |
| Cingulate gyrus | 23 | 4.79 | 21 | -9 | 39 |
| **Left lentiform nucleus** | **18** | **4.49** | **-21** | **-9** | **-6** |
| Pons | 75 | 4.45 | -9 | -33 | -42 |
| **Right hippocampus** | **16** | **4.26** | **36** | **-15** | **-15** |
| **IG < HC** |  |  |  |  |  |
| Left hippocampus | 209 | 6.33 | -33 | -24 | -9 |
| Midbrain | 149 | 6.08 | 9 | -12 | -15 |
| Pons | 172 | 5.90 | -12 | -30 | -39 |
| Cingulate gyrus | 205 | 4.96 | -15 | 18 | 24 |
| **EG > HC** |  |  |  |  |  |
| Left/Right caudate | 221 | 6.63 | 12 | -3 | 18 |
| **IG > HC** |  |  |  |  |  |
| Right caudate | 86 | 6.03 | 12 | -3 | 18 |
| Left caudate | 32 | 4.68 | -12 | -9 | 21 |
| **L3** |  |  |  |  |  |
| **EG > HC** |  |  |  |  |  |
| Right caudate | 60 | 5.72 | 12 | 6 | 18 |
| Left caudate | 69 | 5.34 | -9 | -3 | 21 |
| **IG > HC** |  |  |  |  |  |
| Right caudate | 29 | 4.62 | 9 | -3 | 18 |
| Left caudate | 14 | 4.34 | -12 | -12 | 21 |
| **L4** |  |  |  |  |  |
| **EG < HC** |  |  |  |  |  |
| Left hippocampus | 61 | 5.83 | -36 | -12 | -18 |
| Midbrain | 47 | 5.01 | -9 | 3 | -9 |
| Cingulate gyrus | 92 | 4.91 | -18 | -30 | 33 |
| Midbrain | 36 | 4.38 | 21 | -6 | -6 |
| **Left lentiform nucleus** | **11** | **4.15** | **-21** | **-9** | **-6** |
| **IG < HC** |  |  |  |  |  |
| Left hippocampus | 220 | 6.90 | -33 | -21 | -12 |
| Midbrain | 107 | 5.90 | 9 | -9 | -15 |
| Cingulate gyrus | 136 | 5.13 | -15 | 3 | 33 |
| **EG > HC** |  |  |  |  |  |
| **Left/Right thalamus** | **211** | **6.58** | **12** | **-3** | **18** |
| **IG > HC** |  |  |  |  |  |
| Right caudate | 52 | 5.18 | 9 | -3 | 18 |
| **L5** |  |  |  |  |  |
| **EG** **< HC** |  |  |  |  |  |
| Cingulate gyrus | 187 | 5.61 | -18 | -30 | 33 |
| Left hippocampus | 38 | 5.36 | -36 | -12 | -18 |
| Midbrain | 38 | 5.27 | 21 | -9 | -6 |
| Midbrain | 50 | 5.02 | -6 | -9 | -15 |
| **Left lentiform nucleus** | **15** | **4.41** | **-21** | **-9** | **-6** |
| **Right hippocampus** | **12** | **4.21** | **36** | **-15** | **--18** |
| Pons | 56 | 4.18 | -9 | -33 | -42 |
| **IG < HC** |  |  |  |  |  |
| Pons | 193 | 6.26 | -9 | -27 | -42 |
| Left hippocampus | 194 | 5.98 | -36 | -24 | -9 |
| Midbrain | 133 | 5.86 | 9 | 0 | -9 |
| Cingulate gyrus | 247 | 5.03 | -9 | -3 | 30 |
| **EG > HC** |  |  |  |  |  |
| Right/Left caudate | 223 | 6.55 | 12 | -3 | 18 |
| **IG > HC** |  |  |  |  |  |
| Right caudate | 91 | 5.99 | 12 | -3 | 18 |
| Left caudate | 39 | 4.89 | -12 | 6 | 18 |
| **R1** |  |  |  |  |  |
| **EG < HC** |  |  |  |  |  |
| Left hippocampus | 32 | 5.33 | -36 | -12 | -18 |
| Cingulate gyrus | 55 | 4.09 | -15 | -3 | 33 |
| **IG < HC** |  |  |  |  |  |
| Left hippocampus | 65 | 6.34 | -36 | -12 | -18 |
| Cingulate gyrus | 109 | 5.71 | -15 | 6 | 33 |
| **Right inferior frontal gyrus** | **75** | **4.97** | **54** | **30** | **21** |
| **EG > HC** |  |  |  |  |  |
| Right caudate | 69 | 5.98 | 12 | 0 | 18 |
| **IG > HC** |  |  |  |  |  |
| Right caudate | 26 | 4.49 | 9 | -3 | 18 |
| **R3** |  |  |  |  |  |
| **EG > HC** |  |  |  |  |  |
| **Left caudate** | **74** | **5.70** | **-9** | **-3** | **18** |
| **R4** |  |  |  |  |  |
| **EG < HC** |  |  |  |  |  |
| Left hippocampus | 39 | 5.34 | -36 | -12 | -18 |
| Cingulate gyrus | 17 | 5.16 | -18 | -27 | 33 |
| Cingulate gyrus | 91 | 4.47 | -15 | -6 | 33 |
| Left lentiform nucleus | 10 | 4.32 | -21 | -6 | -6 |
| Pons | 11 | 3.55 | 3 | -18 | -33 |
| **IG < HC** |  |  |  |  |  |
| Left lentiform nucleus/Left hippocampus | 276 | 6.41 | 9 | -9 | -15 |
| Cingulate gyrus | 141 | 5.36 | -15 | -3 | 36 |
| Pons | 83 | 4.99 | -12 | -30 | -39 |
| Cingulate gyrus | 25 | 4.17 | 9 | -15 | 27 |
| **EG > HC** |  |  |  |  |  |
| Left caudate | 96 | 6.50 | -12 | 0 | 18 |
| Right caudate | 75 | 6.31 | 12 | -3 | 18 |
| **IG > HC** |  |  |  |  |  |
| Right caudate | 33 | 4.74 | 9 | -6 | 18 |
| Left caudate | 19 | 4.55 | -12 | 6 | 18 |
| **R5** |  |  |  |  |  |
| **EG < HC** |  |  |  |  |  |
| Cingulate gyrus | 119 | 5.32 | -18 | -30 | 36 |
| Pons | 68 | 4.83 | -12 | -9 | -45 |
| Left hippocampus | 15 | 4.61 | -36 | -12 | -18 |
| Cingulate gyrus | 19 | 4.18 | 24 | -6 | 39 |
| **IG < HC** |  |  |  |  |  |
| Midbrain | 237 | 6.31 | 9 | -9 | -12 |
| Left hippocampus | 63 | 6.21 | -36 | -12 | -18 |
| Cingulate gyrus | 320 | 5.88 | -15 | 6 | 33 |
| **Left supramarginal gyrus** | **66** | **5.83** | **-60** | **-51** | **30** |
| Pons | 163 | 5.41 | 12 | -30 | -39 |
| **EG > HC** |  |  |  |  |  |
| Right caudate | 57 | 5.88 | 12 | -3 | 18 |
| Left caudate | 75 | 5.76 | -6 | -3 | 15 |
| **IG > HC** |  |  |  |  |  |
| Right caudate | 41 | 4.96 | 9 | 3 | 15 |
| Left caudate | 30 | 4.59 | -12 | 6 | 18 |
| **R6** |  |  |  |  |  |
| **EG < HC** |  |  |  |  |  |
| Right middle occipital gyrus | 67 | 5.18 | 48 | -84 | 9 |
| **Right cuneus** | **9** | **3.83** | **27** | **-93** | **30** |
| **IG < HC** |  |  |  |  |  |
| Right middle occipital gyrus | 101 | 5.30 | 45 | -84 | 12 |

Note: The threshold was set at a p < 0.05 (family wise error corrected). EG, effective group; IG, ineffective group, HC, healthy control.
