# Supplementary material for: Multimodal quantitative magnetic resonance imaging of the thalamus in tinnitus patients with different outcomes after sound therapy
Source: CNS Neurosci Ther. 2023 Jun 30;29(12):4070–81. doi: 10.1111/cns.14330 (PMC10651975; doi:10.1111/cns.14330)
Supplement: Supplementary file 3 — Table S2. [file CNS-29-4070-s001.docx]

**Supplementary table 2. Results of ROC curve analysis of the thalamic** **subregions' structural and functional properties as prognostic indicators**

| Thalamic subregions' properties | AUC | sensitivity | specificity | p value | cutoff | PPV (%) | NPV (%) |
| --- | --- | --- | --- | --- | --- | --- | --- |
| L lentiform nucleus | 0.504 | 0.781 | 0.357 | 0.953 | 0.138 | 51.5 | 65.1 |
| R hippocampus | 0.565 | 0.594 | 0.607 | 0.390 | 0.201 | 56.8 | 63.0 |
| L lentiform nucleus | 0.522 | 0.625 | 0.536 | 0.767 | 0.161 | 54.2 | 62.1 |
| L/R thalamus | 0.625 | 0.75 | 0.536 | 0.097 | 0.286 | 58.7 | 71.1 |
| L lentiform nucleus | 0.504 | 0.75 | 0.429 | 0.953 | 0.179 | 53.4 | 66.2 |
| R hippocampus | 0.511 | 1 | 0.143 | 0.882 | 0.143 | 50.5 | 100 |
| R inferior frontal gyrus | **0.675** | 0.438 | 0.964 | **0.02*** | 0.402 | 40.6 | 47.1 |
| L caudata | 0.566 | 0.375 | 0.821 | 0.382 | 0.196 | 64.8 | 60.0 |
| L supramarginal gyrus | **0.770** | 0.781 | 0.714 | **0.000*** | 0.495 | 70.4 | 78.9 |
| R cuneus | 0.574 | 0.594 | 0.607 | 0.328 | 0.201 | 56.8 | 63.0 |
| FA_motor_L | 0.577 | 0.25 | 0.929 | 0.307 | 0.179 | 75.3 | 58.6 |
| FA_prefrontal_R | 0.544 | 0.688 | 0.5 | 0.563 | 0.188 | 54.7 | 64.8 |
| Combination | **0.887** | 0.844 | 0.893 | **0.000*** | 0.737 | 87.4 | 86.7 |

Note: ROC, receiver operator characteristic; L, left; R, right; FA, fractional anisotropy; AUC, area under curve; PPV, positive predictive value; NPV, negative predictive value; *means the p-value < 0.05.
